# Supplementary material for: Dissecting neuron-specific functions of circadian genes using modified cell-specific CRISPR approaches
Source: Proc Natl Acad Sci U S A. 2023 Jul 10;120(29):e2303779120. doi: 10.1073/pnas.2303779120 (PMC10629539; doi:10.1073/pnas.2303779120)
Supplement: Supplementary file 1 — Appendix 01 (PDF) [file pnas.2303779120.sapp.pdf]

PNAS

Supporting Information for

Dissecting neuron-specific functions of circadian genes using modified cell-specific CRISPR approaches.

Shlesha Richhariya[1], Daniel Shin[1], Jasmine Quynh Le[1] and Michael Rosbash[1]*

[1]Howard Hughes Medical Institute, Brandeis University, Waltham, MA 02454

*Correspondence – rosbash@brandeis.edu

This PDF file includes:

Supporting Materials and Methods
Figures S1 to S8
Tables S1 to S4
Legend for Dataset S1
SI References

Other supporting materials for this manuscript include the following:

Dataset S1

SI Materials and Methods:

Generation of Fly Lines

To generate lines expressing 3X-guides under UAS-control, we used previously described protocols (1, 2). Briefly, gRNAs specific to the target genes were identified using the CRISPR Optimal Target Finder tool (3). These gRNA sequences were incorporated into the tRNA backbone using 2 PCR reactions, primers for which are listed in supplementary Table S4. The PCR products were assembled into the UASpCFD6 vector (Addgene #73915) digested with BbsI-HF enzyme (NEB) using Gibson assembly. Sequence verified plasmids were injected into the attP2 site on the third chromosome by Rainbow Transgenic Flies Inc (Camarillo, CA, USA). Positive transformants were screened for by red eye color based on the miniwhite marker.

Locomotor Activity and Sleep Behavior

Circadian rhythmicity and sleep analysis was performed using the 2020 version of Sleep and Circadian Analysis MATLAB Program (SCAMP) developed by Christopher G. Vecsey (4). For period analysis, only rhythmic flies (Rhythmicity Index > 0.25) were used, apart from PDF perturbations in Fig. S6 and Table S2, where flies with Rhythmicity Index >0.2 were defined as rhythmic to look for residual short period rhythms in partially arrhythmic flies. E-peak timing was calculated as the highest point in bins from ZT6.5-ZT10.5 of average activity in 30 min bins exported from SCAMP. Sleep structure analysis for p-wake and p-doze (5) was also performed using this new version of SCAMP.

For phase shift experiments about 2-week-old males flies were entrained to LD for at least 3 days and on the last night of LD, 1 hour light pulse (500 lux) was delivered either at ZT15, ZT18 or ZT21. For one set of flies, no light pulse was given, and these were used as controls to calculate phase shift using the SCAMP program. Average shifts from days 3-5 are reported.

Heat shock for inducible Cas9

For experiments with the inducible Cas9 system, 2-5 days old male flies of the appropriate genotype were heat shocked on 3 consecutive days. The first heat shock was 25 mins, and the two subsequent ones were 40 mins each. Heat shock was delivered by placing flies in an empty food vial and submerging the vial in a water bath set at 37°C. Flies were removed promptly and allowed to recover briefly at room temperature before being transferred to a fresh vial with standard cornmeal food. Once the flies recovered completely, the flies were placed in an incubator set to 25°C. Flies were aged for 2 weeks from the day of the first heat shock before loading them in behavior experiments. Control flies were collected at the same time and aged simultaneously but without the heat shock.

Auxin feeding for adult-specific behavior with AGES

For AGES experiments, 2-5 days old male flies were fed Instant *Drosophila* Medium (Formula 424®, Carolina) reconstituted either in 10mM Auxin (GK2088, Glentham Life Sciences) or distilled water (for controls). Flies were fed this food for about 2 weeks before loading them in behavior tubes with standard behavior food (4% sucrose and 2% agar). Since CRISPR based mutagenesis irreversibly alters the DNA in the cells of interest, we used standard behavior food for behavior analysis post auxin feeding to make experiments simpler. For AGES time course, 0-2 day old male flies expressing gRNA and Cas9 with CLK856 Gal4 and AGES were collected and divided into 4 groups – No auxin, auxin fed for 2days, 4 days and 7 days. Flies were fed auxin for the indicated duration, starting later for shorter durations, and then tested for behavior on standard behavior food.

Immunohistochemistry and Image Analysis

Whole flies of the same genotype and age as the behavior experiment cohort were fixed at ZT18-19 in 4% paraformaldehyde in PBS with 0.4% Triton X-100 for 2 hours and 30 minutes at room temperature. Tubes were wrapped in tin foil to protect them from light while rotating on a shaker. Flies were then washed three times (10 minutes each) with 0.4% PBST before dissecting the

brain. Dissected brains were washed and blocked with 10% Normal Goat Serum (NGS; Jackson labs) in 0.4% PBST (blocking buffer) for 2 hours at room temperature or at 4°C overnight. The following primary antibodies were used diluted in blocking buffer– chicken anti-GFP (Abcam ab13970; 1:1000), mouse anti-PDF (DSHB-PDF C7; 1:1000), guinea pig anti-VRI (gift of Paul Hardin; 1:3000 (6)), rat anti-TIM (Rosbash lab; 1:250, (7)), rabbit anti-PER (Rosbash lab, 1:1000, (7)), rabbit anti-dsRed (Takara Bio- 632393; 1:300) and rat anti-RFP (Proteintech-5f8; 1:1000). Primary antibody was incubated for 24-36 hours at 4°C. The brains were then washed three times (30 minutes each) in 0.4% PBST, and corresponding secondary antibodies were used at a 1:500 dilution in blocking buffer and incubated at 4°C overnight. Brains were again washed three times (30 minutes each) in 0.4% PBS, mounted in VectaShield mounting medium (Vector Laboratories).

For CRY immunostaining, flies were kept in constant darkness for several days to allow accumulation and detection of CRY as described earlier (8). Flies were fixed at CT0-1 (DD4) in dark conditions. The rat anti-CRY antisera (9) were incubated with fixed heads of the null mutants, cry^{01} (10) to reduce background staining. This primary rat anti-CRY antibody was used at 1:200.

Images were acquired using Leica Stellaris 8 confocal microscope equipped with a white light laser. Images for one set of experiments were acquired using the Leica SP5 confocal microscope. For each experiment, the laser settings were kept constant across genotypes. Either 20X air objective or a 40X oil objective (for image quantification) with NA of 1.43 was used. Image analysis was performed with Fiji (11) using the Time Analyzer V3 plugin to mark ROIs across channels and measure intensity of the PDF channel. Background intensity for a z-stack was measured from three random background regions, averaged, and subtracted from each ROI.

Data representation and Statistical Analysis

Some of the figure panels (Fig. S5A, S7A and S8A) were created using BioRender. Boxplots were generated using BoxPlotR (12). For all boxplots, center lines show the medians; box limits

indicate the 25th and 75th percentiles as determined by R software; whiskers extend 1.5 times the interquartile range from the 25th and 75th percentiles, outliers are represented by dots. Source data for all figures are included in SI Dataset 1. Statistical tests performed are described in the figure legends. All statistical tests were performed using the free online tool Statistics Kingdom https://www.statskingdom.com/index.html

Supplementary Figures

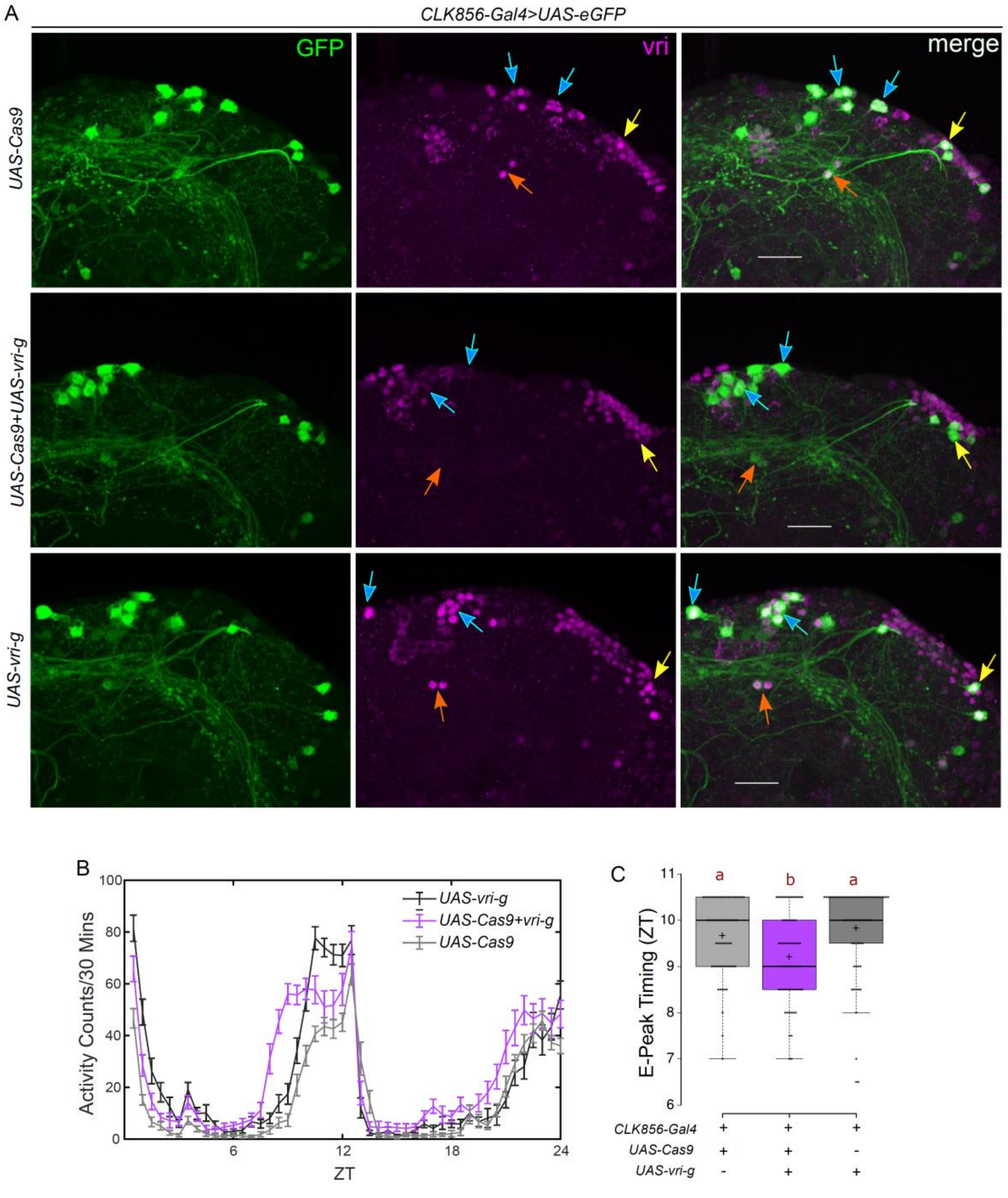

Fig. S1: Loss of VRI from clock neurons leads to an advanced evening peak. A. Representative images of the dorsal circadian neurons stained for GFP and VRI at ZT19. There is loss of VRI staining in cells expressing *CLK856-Gal4* (marked by GFP) but no loss in GFP-negative cells. Arrows point to examples of Gal4-positive DN1s (blue), DN2s (red) and DN3s (yellow) in the indicated genotypes. Scale bar represents 20µm. **B**. Average activity

counts for flies in an experiment plotted as a function of time. Flies with loss of VRI in *CLK856-Gal4* labelled neurons showed an advanced evening peak. **C**. Quantification of the evening peak (E-peak) timing from individual flies, n ≥ 62 per genotype from at least two independent experiments. Letters represent statistically distinct groups; $p<0.01$, Kruskal Wallis test followed by a post hoc Dunn's test.

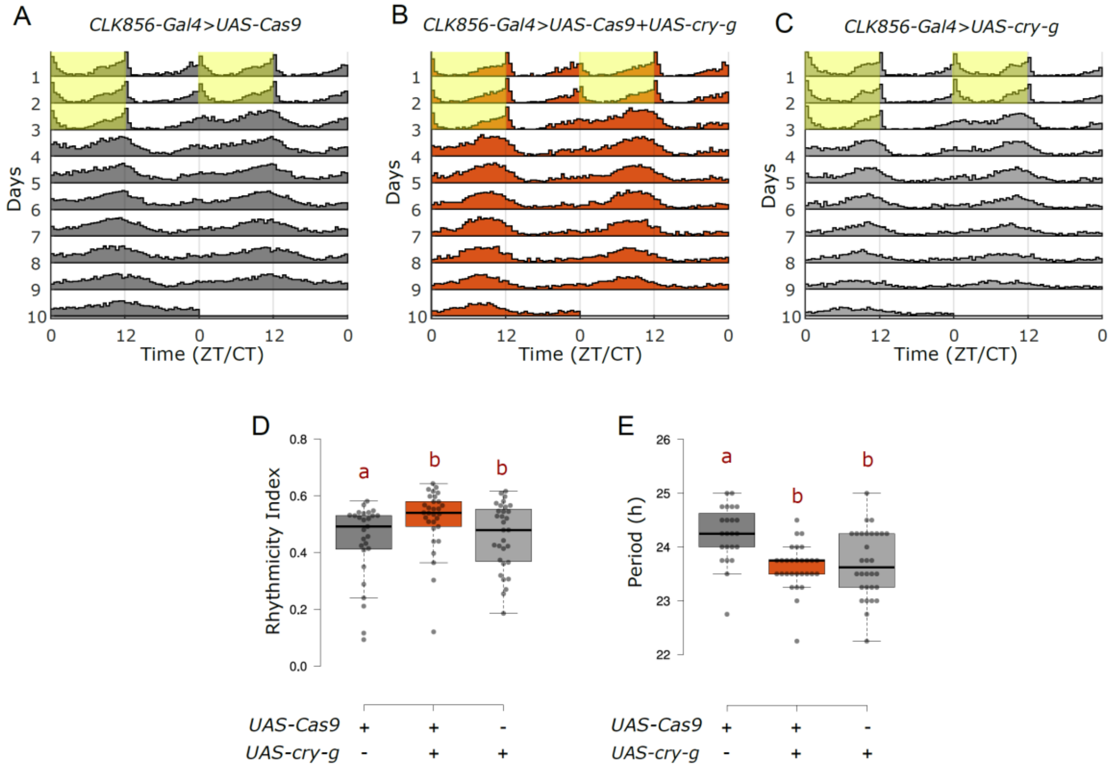

Fig. S2: CRY mutation in clock neurons does not affect circadian behavior under constant darkness. A-C. Actograms represent double-plotted average activity of flies from an experiment across multiple days. Yellow panels indicate lights ON. **D**. Rhythmicity Index for individual flies quantified for DD2-7 represented by a boxplot, n ≥ 27 per genotype, letters represent statistically distinct groups, p<0.01, Mann-Whitney U test post Kruskal Wallis ANOVA. **E**. Free running period under constant darkness for individual flies quantified for DD2-7 represented by a boxplot, letters represent statistically distinct groups; p<0.01, Kruskal Wallis test followed by a post hoc Dunn’s test.

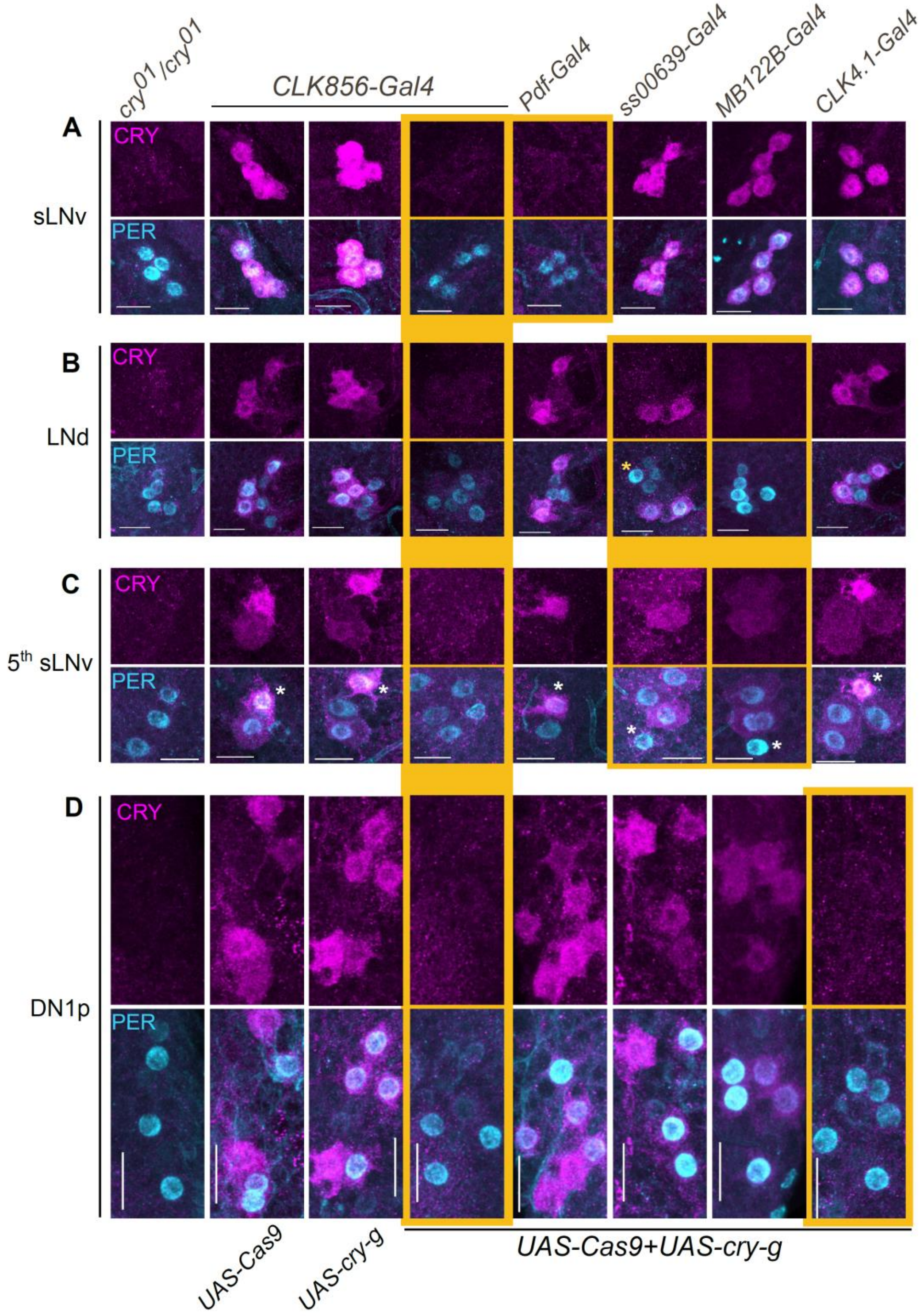

Fig. S3: CRISPR mediated cell specific mutagenesis of cry leads to cell-type specific loss of CRY-staining. Images from brains co-stained with CRY and PER, to label clock neurons at CT0-1 (DD4). In controls (columns 2 and 3), CRY staining is detected strongly in

the sLNvs, 3 of the 6 LNds, the 5th sLNv and a subset of the DN1ps whereas weakly in the lLNvs. CRY staining was absent from all clock neurons in the cry null mutant (*cry*^{01}/*cry*^{01}; column 1). Similarly, CRISPR mediated knockout of CRY with *CLK856-Gal4* led to severely reduced CRY staining in all clock neurons (A-D; column 4), whereas CRISPR mutagenesis with specific Gal4s led to loss of CRY staining from specific neurons as described below. Panels where CRY staining is perturbed are highlighted in yellow. **A**. CRY staining in the sLNv neurons was perturbed only with *Pdf-Gal4.* **B**. CRY mutagenesis with *MB122B-Gal4* led to loss of CRY staining from all three LNds whereas that with *ss00639-Gal4* led to loss of CRY staining from only one LNd (marked by asterisk). **C.** In the 5th sLNv (marked by asterisk), CRY staining is lost with CRISPR mutagenesis in *MB122B-Gal4* and *ss00639-Gal4.* **D**. CRY staining in the DN1ps is lost only when expression is targeted with the *CLK4.1-Gal4.* Scale bars represent 10µm.

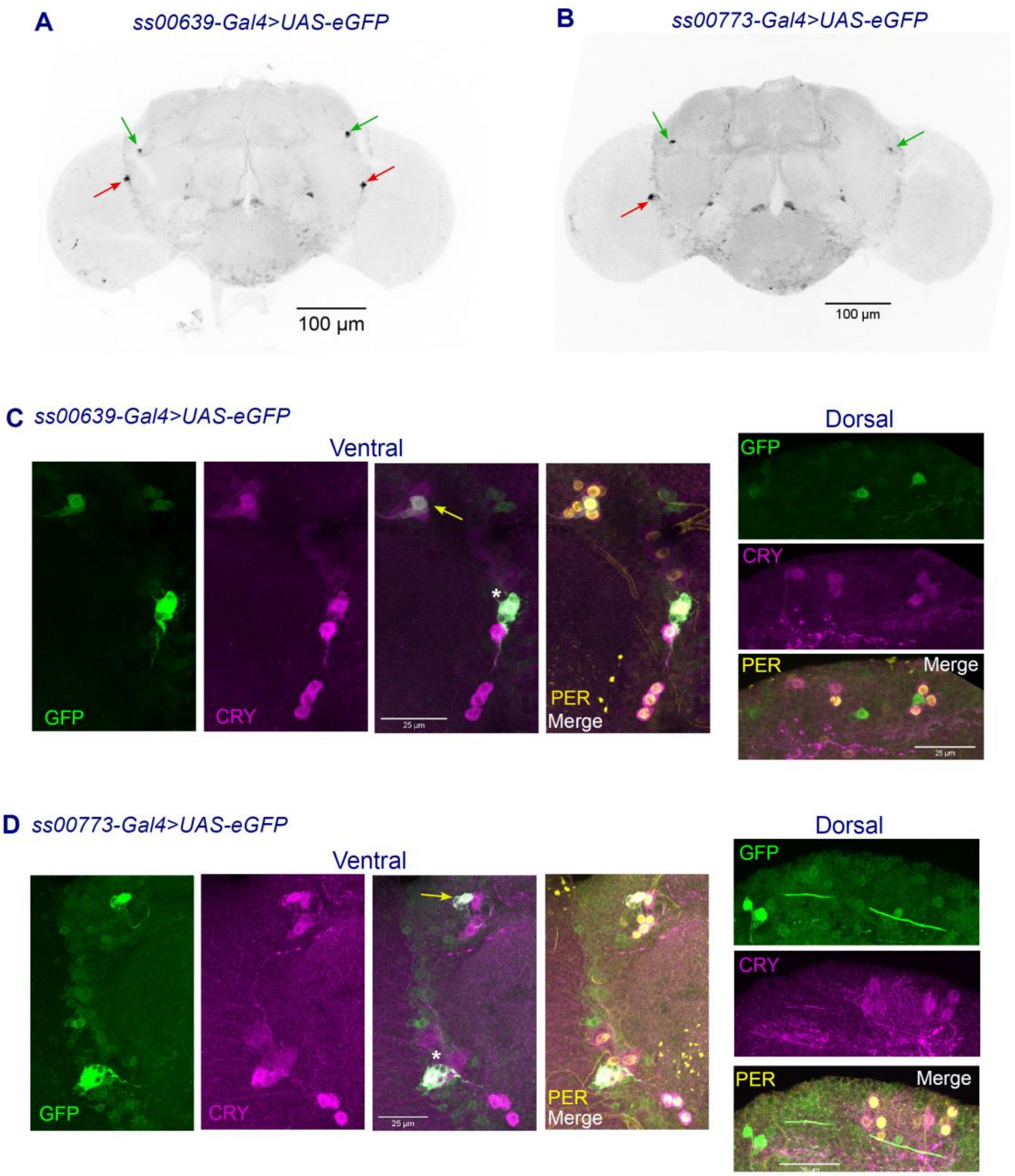

Fig. S4: Split-Gal4 drivers labelling a subset of evening cells. A-B: Full brain expression patterns of the two split-Gal4 drivers labelled by UAS-eGFP. **C-D:** Split-Gal4 lines co-stained with CRY and PERIOD (PER) to label clock neurons. Both split-Gal4s label the CRY and PER positive 5th sLNv (marked by asterisk) and one LNd (yellow arrow). In panels A-B, red arrows point to the 5th-sLNv and green arrows to an LNd neuron as identified by co-staining with clock

proteins in C-D. The *ss00639-Gal4* labels the 5th-sLNv + 1 LNd per hemisphere in all brains. The *ss00773-Gal4* also labels the 5th-sLNv + 1 LNd, but it does so more variably and labels about 2-4 of these clock cells per brain. Both Gal4s also label a few extra cells, *ss00773-Gal4* more so than *ss00639-Gal4,* but none of them are either PER or CRY positive.

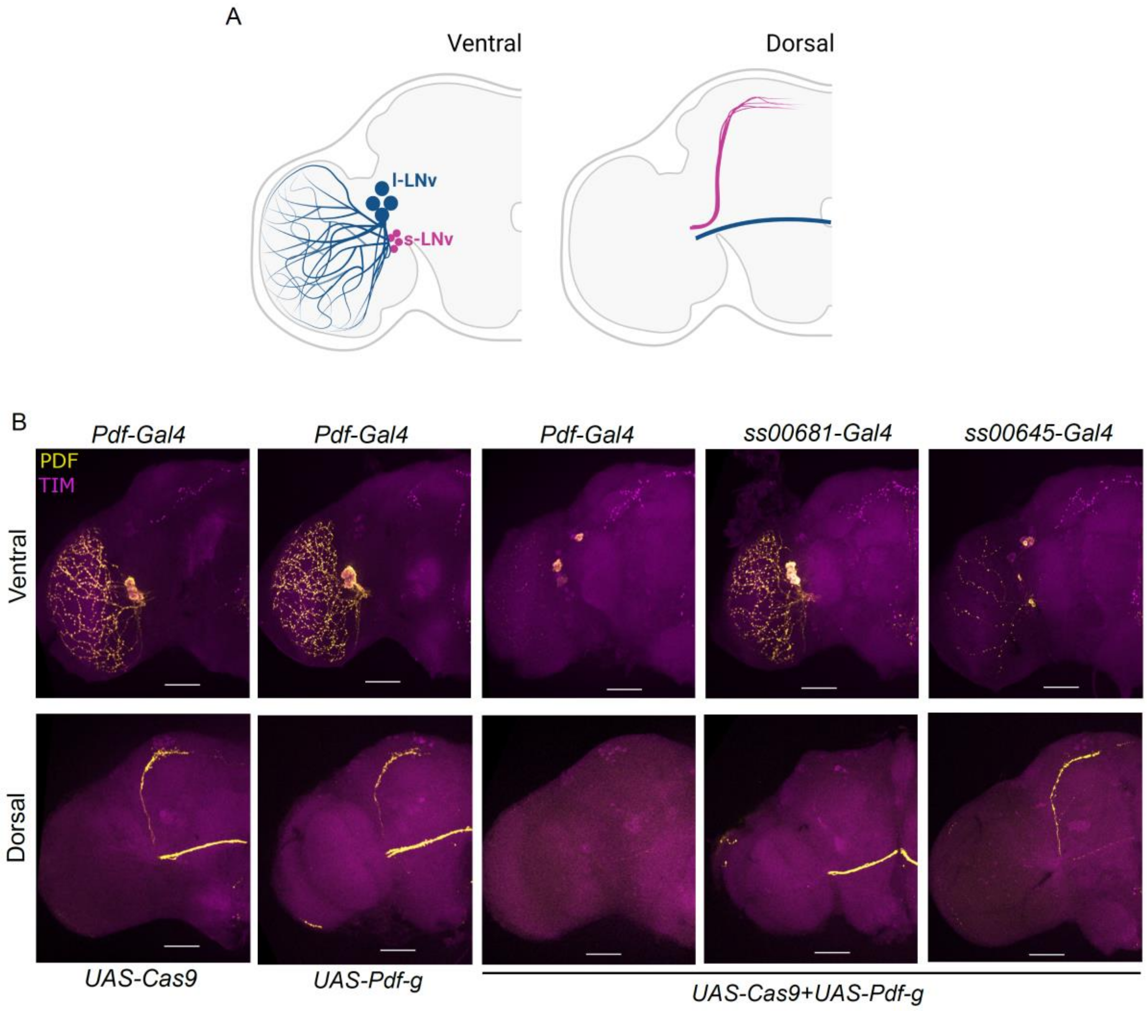

Fig. S5: CRISPR strategy successfully eliminates PDF staining from cell type-specific projections. A. Cartoon representation of the projection patterns of small (magenta) and large (blue) LNV neurons in the ventral and dorsal *Drosophila* brain. **B**. Representative images of hemibrains imaged from the ventral (top) or dorsal (bottom) side stained for PDF and TIM. For the dorsal panels, brightness-contrast was enhanced for the PDF channel equally across genotypes to confirm absence of PDF staining from the respective processes. Expression of both *UAS-Cas9* and *UAS-Pdf-g* with *Pdf-Gal4* leads to loss of all PDF staining from all PDF neurons. Expression with *ss00681-Gal4* that labels only the sLNvs leads to loss of PDF staining from their cell bodies and projections with no obvious effect on PDF staining in lLNvs.

Expression with *ss00645-Gal4* leads to loss of PDF staining in most projections of these neurons with no obvious effect on PDF staining in sLNvs.

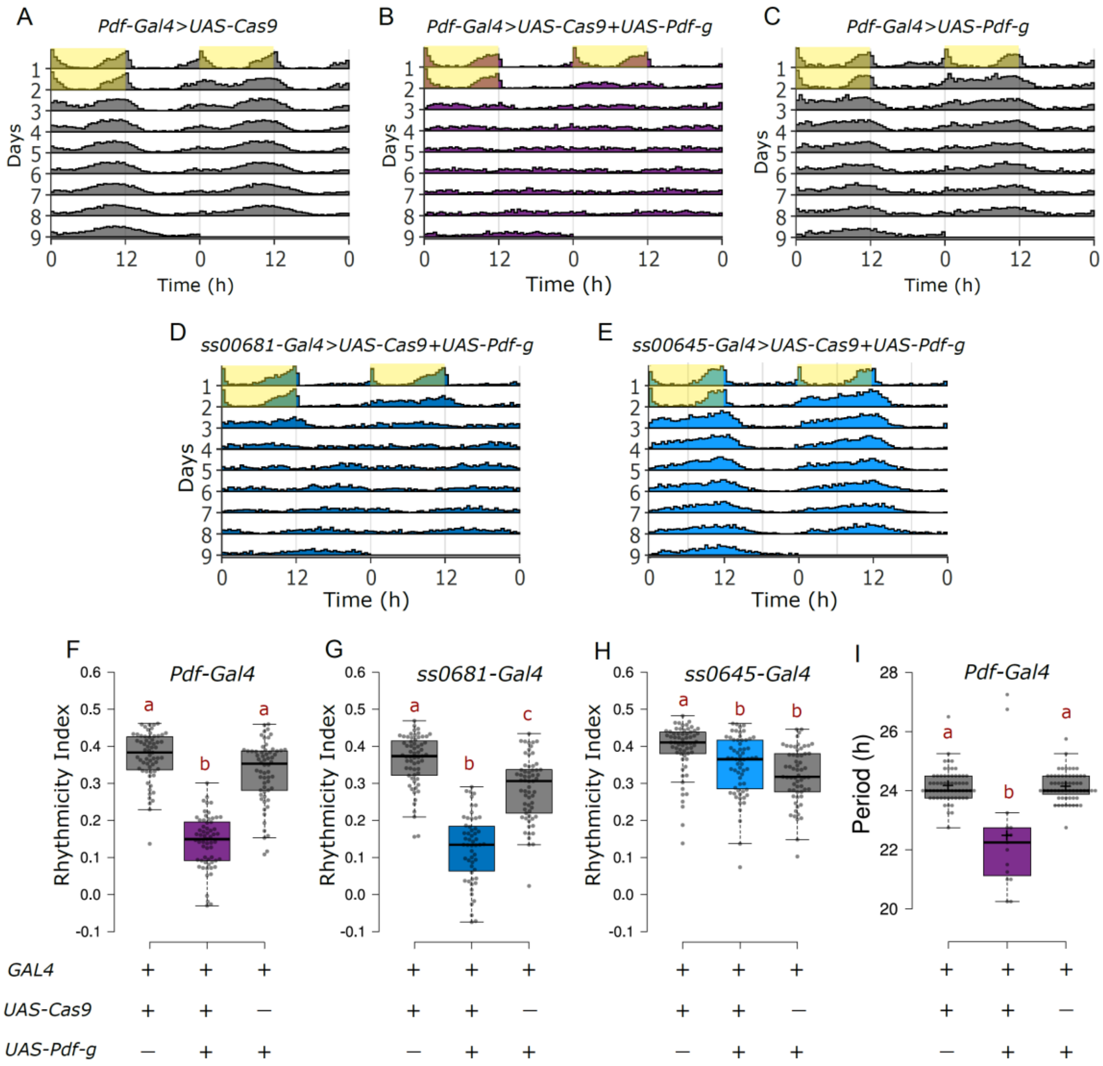

Fig. S6: Loss of PDF from sLNvs leads to loss of rhythmicity under constant darkness. **A-E**. Actograms represent double-plotted average activity of flies from an experiment across multiple days. Yellow panels indicate lights ON. **F-H**. Rhythmicity Index for individual flies quantified for DD1-4 represented by a boxplot, n ≥ 55 per genotype from at least two independent experiments, letters represent statistically distinct groups; p<0.01, Kruskal Wallis test followed by a post hoc Dunn's test. Loss of PDF from both sLNvs and lLNvs or from sLNvs alone causes arrhythmicity in constant darkness, whereas loss of PDF from lLNvs alone has no effect. **I.** Free running period for flies of the indicated genotypes, rhythmic flies (RI>0.2) with loss of PDF in PDF neurons have a shorter period.

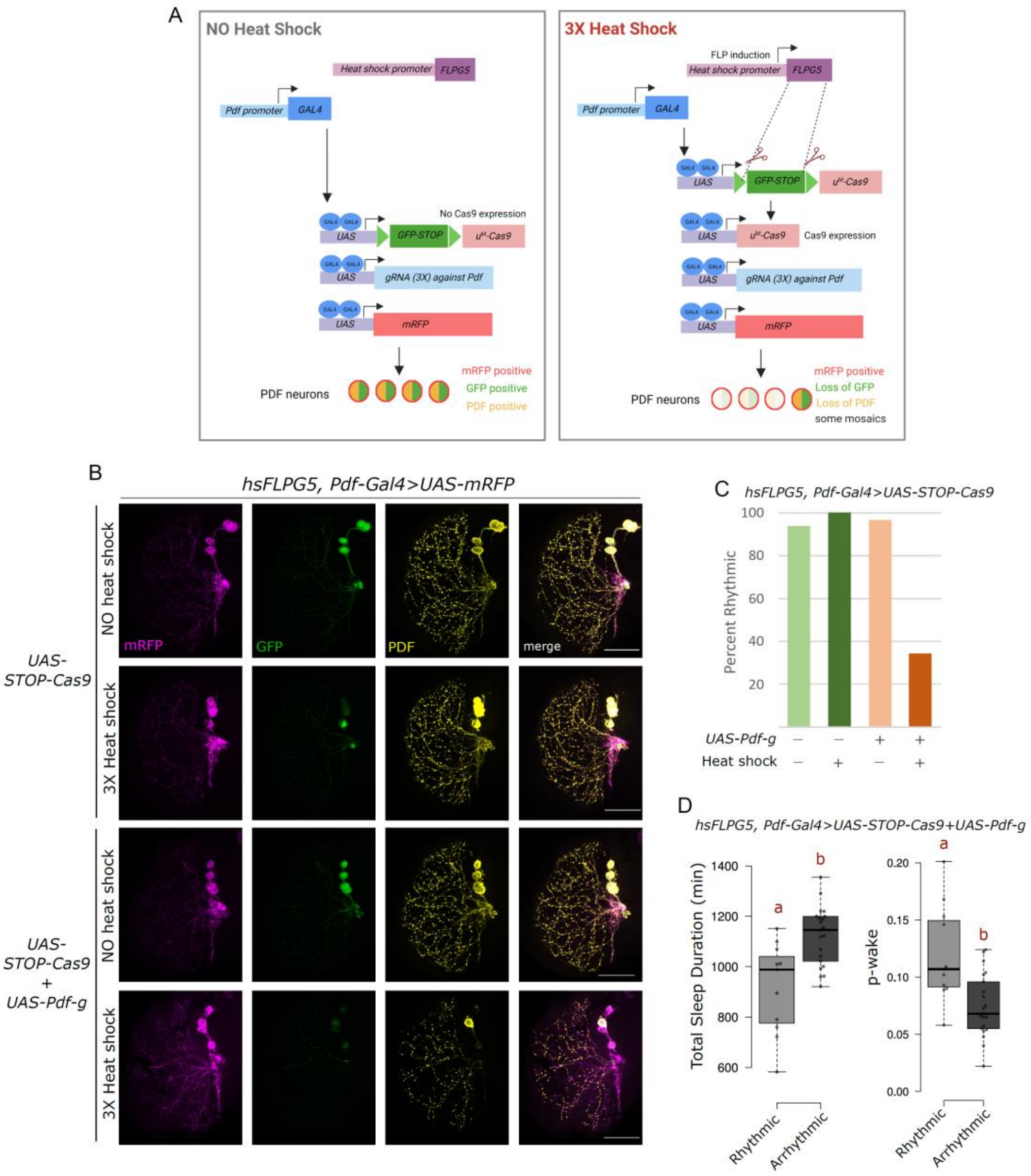

Fig. S7: The inducible-Cas9 systems is an effective tool for adult specific and cell type specific CRISPR mutagenesis despite some mosaics. A. Cartoon representation of the inducible Cas9 system (Port et. al., 2020) adapted to the adult-specific perturbation of PDF from PDF neurons. **B**. Representative images of the optic lobe ventral projections of the lLNvs. Scale bar represents 50µm. **C**. Plot indicating the percentage of flies rhythmic under the indicated conditions. **D**. Sleep parameters of flies expressing both *UAS-Cas9* and *UAS-Pdf-g* with heat-shock separated into rhythmic (RI>0.25) and arrhythmic (RI<0.25) categories, n=11 for rhythmic and 21 for arrhythmic. Arrhythmic flies have more sleep and a lower p-wake;

p<0.01, Mann Whitney U test. These rhythmic flies could be the result of the mosaicism, i.e., PDF expression in 1-2 sLNvs might be sufficient for quasi-normal circadian and sleep behavior.

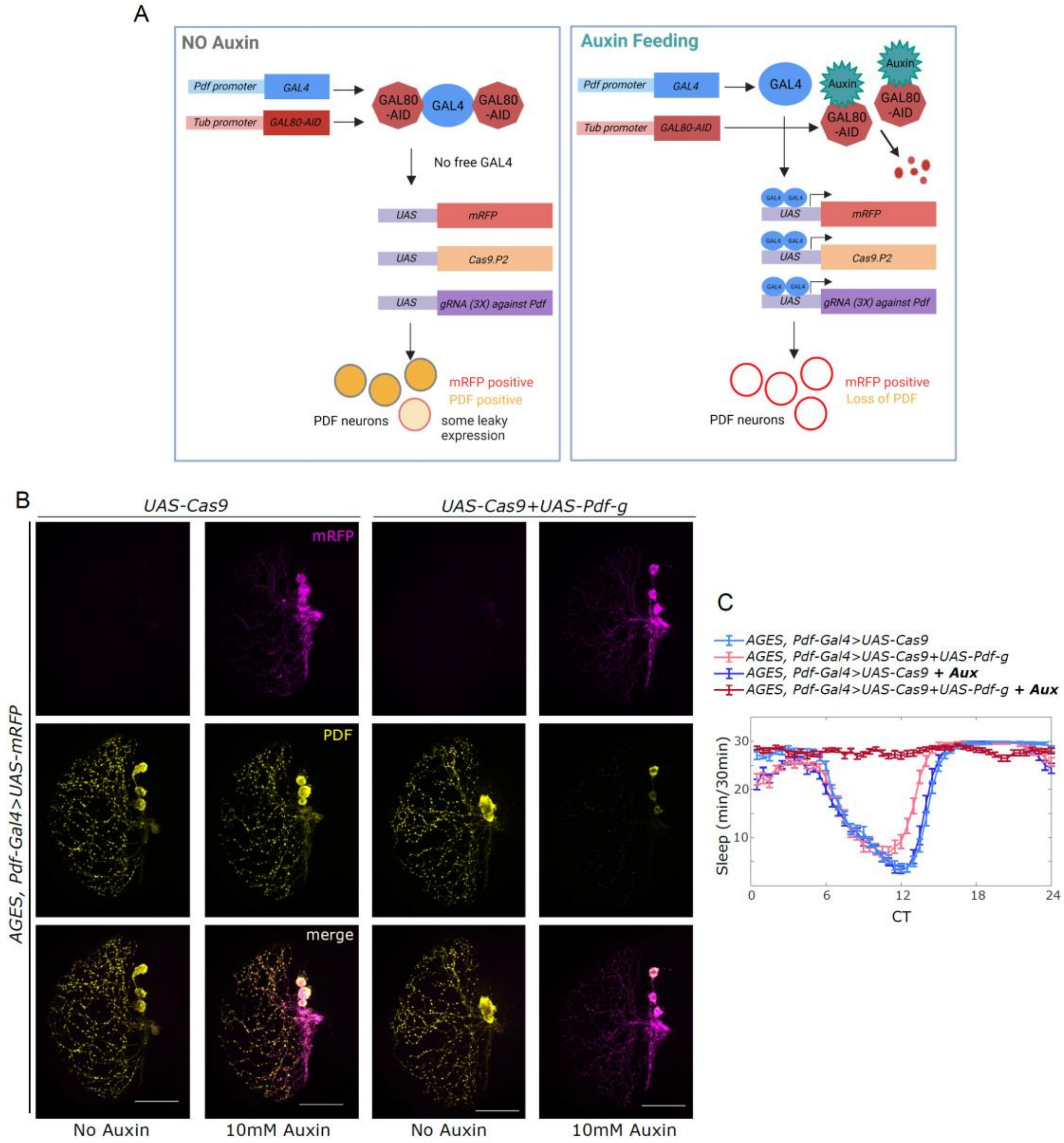

Fig. S8: AGES is mildly leaky, but still an effective tool for adult-specific, cell type-specific CRISPR mutagenesis. A. Cartoon representation of the AGES system (McClure et. al., 2022) adapted to the adult specific perturbation of PDF from PDF neurons. **B**. Representative images of the optic lobe ventral projections of the lLNvs. Scale bar represents 50µm. **C**. Sleep plot representing average sleep of flies from DD1-4 in 30-minute bins.

Supplementary Tables

Table S1: Summary of circadian behavior with cell type-specific CRY mutants in constant light.

Genotype	Cells labelled	n	Rhythmicity Index LL2-7 (Mean ± SEM)	%Rhythmic (RI>0.25)	Period (h) (Mean ± SEM)
CLK856-Gal4>UAS-Cas9	Most clock neurons (many DN3s missing)	85	0.13 ± 0.01	10.6	-
CLK856-Gal4>UAS-CRYg		72	0.11 ± 0.01	6.9	-
CLK856-Gal4>UAS-Cas9+UAS-CRYg		**92**	**0.40 ± 0.01**	**90.2**	**24.71 ± 0.07**
PDF-Gal4>UAS-Cas9	sLNvs+ lLNvs	118	0.10 ± 0.01	6.8	-
PDF-Gal4>UAS-CRYg		119	0.12 ± 0.01	5.0	-
PDF-Gal4>UAS-Cas9+UAS-CRYg		**121**	**0.17 ± 0.01**	**14.9**	-
dvPDF-Gal4>UAS-Cas9	sLNvs+ lLNvs+ 5th sLNv+ 1 CRY^{+} ITP^{+} LNd + 3 CRY^{-}LNds	56	0.14 ± 0.01	8.9	-
dvPDF-Gal4>UAS-CRYg		51	0.15 ± 0.01	9.8	-
dvPDF-Gal4>UAS-Cas9+UAS-CRYg		**58**	**0.52 ± 0.02**	**91.4**	**24.24 ± 0.06**
MB122B-Gal4>UAS-Cas9	5th sLNv + 1 $CRY^{+}ITP^{+}$ + 2 CRY^{+} $Trissin^{+}$ LNds	64	0.15 ± 0.01	9.4	-
MB122B--Gal4>UAS-CRYg		61	0.14 ± 0.01	3.3	-
MB122B-Gal4>UAS-Cas9+UAS-CRYg		**63**	**0.37 ± 0.02**	**79.4**	**25.08 ± 0.08**
ss00639-Gal4>UAS-Cas9	5th sLNv + 1 CRY^{+} LNd	64	0.14 ± 0.01	9.4	-
ss00639-Gal4>UAS-CRYg		58	0.15 ± 0.01	10.3	**-**
ss00639-Gal4>UAS-Cas9+UAS-CRYg		**60**	**0.41 ± 0.01**	**91.7**	**25.11 ± 0.06**
ss00773-Gal4>UAS-Cas9	5th sLNv + 1 CRY^{+} LNd	63	0.14 ± 0.01	4.8	-
ss00773-Gal4>UAS-CRYg		60	0.15 ± 0.01	8.3	-
ss00773-Gal4>UAS-Cas9+UAS-CRYg		**61**	**0.29 ± 0.02**	**60.7**	**25.06 ± 0.14**
CLK4.1-Gal4>UAS-Cas9	DN1p	72	0.12 ± 0.01	9.7	-
CLK4.1-Gal4>UAS-CRYg		69	0.13 ± 0.01	5.8	-
CLK4.1-Gal4>UAS-Cas9+UAS-CRYg		**83**	**0.33 ± 0.01**	**74.7**	**21.82 ± 0.08**

Table S2: Summary of circadian and sleep behavior upon loss of PDF from specific neurons.

Genotype	cells labelled	n	Percent Rhythmic (RI>0.2)	Rhythmicity Index (DD1-4)	Average total sleep per day(min)	Activity while awake	pwake	pdoze
				Mean ± SEM				
PDF-Gal4>UAS-Cas9	All sLNvs + lLNvs	63	98.4	0.37 ± 0.01	914 ± 22	1.62 ± 0.04	0.12 ± 0.006	0.37 ± 0.01
PDF-Gal4>UAS-PDFg		62	90.3	0.33 ± 0.01	873 ± 23	1.79 ± 0.05	0.13 ± 0.007	0.35 ± 0.01
PDF-Gal4>UAS-Cas9 + UAS-PDFg		**61**	**24.6**	**0.14 ± 0.01**	**1178 ± 19**	**2.52 ± 0.10**	**0.06 ± 0.005**	**0.40 ± 0.01**
ss00681-Gal4>UAS-Cas9	All sLNvs	63	96.8	0.36 ± 0.01	924 ± 19	1.81 ± 0.04	0.12 ± 0.006	0.38 ± 0.01
ss00681-Gal4>UAS-PDFg		62	83.9	0.29 ± 0.01	972 ± 21	2.17 ± 0.07	0.12 ± 0.006	0.42 ± 0.01
ss00681-Gal4>UAS-Cas9 + UAS-PDFg		**55**	**23.6**	**0.13 ± 0.01**	**1284 ± 13**	**2.57 ± 0.10**	**0.04 ± 0.003**	**0.44 ± 0.01**
ss00645-Gal4>UAS-Cas9	Variably 3-4 lLNvs per hemi-brain	62	96.8	0.39 ± 0.01	957 ± 23	1.79 ± 0.05	0.11 ± 0.008	0.34 ± 0.01
ss00645-Gal4>UAS-PDFg		61	96.7	0.32 ± 0.01	1068 ± 14	2.37 ± 0.10	0.08 ± 0.004	0.38 ± 0.01
ss00645-Gal4>UAS-Cas9 + UAS-PDFg		**63**	**95.2**	**0.35 ± 0.01**	**1142 ± 15**	**2.20 ± 0.08**	**0.07 ± 0.003**	**0.38 ± 0.1**

Table S3: List of fly strains.

Genotype	Reference/Source	Stock number
CLK856-Gal4	(13);<br>Bloomington Drosophila Stock Center	BDSC 93198
UAS-eGFP	Bloomington Drosophila Stock Center	BDSC 5430
UAS-mRFP	Bloomington Drosophila Stock Center	BDSC 32219
UAS-Cas9.P2 (attP40)	Bloomington Drosophila Stock Center	BDSC 58985
UAS-Cas9.P2 (attP2)	Bloomington Drosophila Stock Center	BDSC 58986
AGES	(14);<br>Bloomington Drosophila Stock Center	BDSC 92470
hsFLPG5	Bloomington Drosophila Stock Center	BDSC 55814
cry^{01}	(10)	NA
PDF-Gal4	(15)	NA
CLK4.1-Gal4	(16, 17)	NA
dvPDF-Gal4	(18)	NA
MB122B-Gal4	(19)	Lines made by Drs. Dione, Nern and Rubin, Janelia Research Campus
ss00681-Gal4	(20)	
ss00645-Gal4	(2)	
ss00639-Gal4	NA	
ss00773-Gal4	NA	
UAS-STOP-Cas9 (UAS-FRT-mEGFP-FRT-uM-Cas9)	(21);<br>Vienna Drosophila Resource Center	VDRC 340008
UAS-*per-g*	(2)	NA
UAS-3X-vrig (attP2)	This study	NA
UAS-3X-cryg (attP2)	This study	NA
UAS-3X-Pdfg (attP2)	This study	NA

Table S4: Primer sequences used for cloning.

name	sequence 5'-> 3'
vri-1	GGCCCGGGTTCGATTCCCGGCCGATGCA**TACAGCCAGCAGAAGCTGAG**GTTTCAGAGCTATGCTGGAAAC
vri-2	**ACTGCGGTCCATTGCCACCC**TGCACCAGCCGGGAATCGAACC
vri-3	**GGGTGGCAATGGACCGCAGT**GTTTCAGAGCTATGCTGGAAAC
vri-4	TTTAACTTGCTATTTCTAGCTCTAAAAC**GACATTGCGGTTTAGTGCCT**TGCACCAGCCGGGAATCGAACC
cry-1	GGCCCGGGTTCGATTCCCGGCCGATGCA**CGCGAGGGGCGAATGTGATT**GTTTCAGAGCTATGCTGGAAAC
cry-2	**CCTGTAGCTGATCATCGATG**TGCACCAGCCGGGAATCGAACC
cry-3	**CATCGATGATCAGCTACAGG**GTTTCAGAGCTATGCTGGAAAC
cry-4	TTTAACTTGCTATTTCTAGCTCTAAAAC**TACACATTGAAGTGCTCCGG**TGCACCAGCCGGGAATCGAACC
Pdf-1	GGCCCGGGTTCGATTCCCGGCCGATGCA**TGCTGCCAGTGGGGATACTG**GTTTCAGAGCTATGCTGGAAAC
Pdf-2	**CGTTGTTGAACCAGTCGAGG**TGCACCAGCCGGGAATCGAACC
Pdf-3	**CCTCGACTGGTTCAACAACG**GTTTCAGAGCTATGCTGGAAAC
Pdf-4	TTTAACTTGCTATTTCTAGCTCTAAAAC**GCATCGTTCATGTTCTTGGG**TGCACCAGCCGGGAATCGAACC

Legend for Supplementary Dataset 1: File contains source data and statistical test for all figures.

SI References

1. F. Port, S. L. Bullock, Augmenting CRISPR applications in Drosophila with tRNA-flanked sgRNAs. *Nat. Methods* **13**, 852–854 (2016).

2. M. Schlichting, M. M. Díaz, J. Xin, M. Rosbash, Neuron-specific knockouts indicate the importance of network communication to Drosophila rhythmicity. *eLife* **8**, e48301 (2019).

3. S. J. Gratz, *et al.*, Highly specific and efficient CRISPR/Cas9-catalyzed homology-directed repair in Drosophila. *Genetics* **196**, 961–971 (2014).

4. N. Donelson, *et al.*, High-Resolution Positional Tracking for Long-Term Analysis of Drosophila Sleep and Locomotion Using the “Tracker” Program. *PLOS ONE* **7**, e37250 (2012).

5. T. D. Wiggin, *et al.*, Covert sleep-related biological processes are revealed by probabilistic analysis in Drosophila. *Proc. Natl. Acad. Sci. U. S. A.* **117**, 10024–10034 (2020).

6. N. R. J. Glossop, *et al.*, VRILLE feeds back to control circadian transcription of Clock in the Drosophila circadian oscillator. *Neuron* **37**, 249–261 (2003).

7. D. Ma, *et al.*, A transcriptomic taxonomy of Drosophila circadian neurons around the clock. *eLife* **10**, e63056 (2021).

8. T. Yoshii, T. Todo, C. Wülbeck, R. Stanewsky, C. Helfrich-Förster, Cryptochrome is present in the compound eyes and a subset of Drosophila’s clock neurons. *J. Comp. Neurol.* **508**, 952–966 (2008).

9. P. Emery, W. V. So, M. Kaneko, J. C. Hall, M. Rosbash, CRY, a Drosophila Clock and Light-Regulated Cryptochrome, Is a Major Contributor to Circadian Rhythm Resetting and Photosensitivity. *Cell* **95**, 669–679 (1998).

10. E. Dolezelova, D. Dolezel, J. C. Hall, Rhythm Defects Caused by Newly Engineered Null Mutations in Drosophila’s cryptochrome Gene. *Genetics* **177**, 329–345 (2007).

11. J. Schindelin, *et al.*, Fiji: an open-source platform for biological-image analysis. *Nat. Methods* **9**, 676–682 (2012).

12. M. Spitzer, J. Wildenhain, J. Rappsilber, M. Tyers, BoxPlotR: a web tool for generation of box plots. *Nat. Methods* **11**, 121–122 (2014).

13. J. O. Gummadova, G. A. Coutts, N. R. J. Glossop, Analysis of the Drosophila Clock Promoter Reveals Heterogeneity in Expression between Subgroups of Central Oscillator Cells and Identifies a Novel Enhancer Region. *J. Biol. Rhythms* **24**, 353–367 (2009).

14. C. D. McClure, *et al.*, An auxin-inducible, GAL4-compatible, gene expression system for Drosophila. *eLife* **11**, e67598 (2022).

15. S. C. Renn, J. H. Park, M. Rosbash, J. C. Hall, P. H. Taghert, A pdf neuropeptide gene mutation and ablation of PDF neurons each cause severe abnormalities of behavioral circadian rhythms in Drosophila. *Cell* **99**, 791–802 (1999).

16. L. Zhang, *et al.*, DN1p Circadian Neurons Coordinate Acute Light and PDF Inputs to Produce Robust Daily Behavior in Drosophila. *Curr. Biol.* **20**, 591–599 (2010).

17. Y. Zhang, Y. Liu, D. Bilodeau-Wentworth, P. E. Hardin, P. Emery, Light and Temperature Control the Contribution of Specific DN1 Neurons to Drosophila Circadian Behavior. *Curr. Biol. CB* **20**, 600–605 (2010).

18. J. H. Bahn, G. Lee, J. H. Park, Comparative Analysis of Pdf-Mediated Circadian Behaviors Between Drosophila melanogaster and D. virilis. *Genetics* **181**, 965–975 (2009).

19. N. Bulthuis, K. R. Spontak, B. Kleeman, D. J. Cavanaugh, Neuronal Activity in Non-LNv Clock Cells is Required to Produce Free-Running Rest:Activity Rhythms in Drosophila. *J. Biol. Rhythms* **34**, 249–271 (2019).

20. X. Liang, T. E. Holy, P. H. Taghert, A Series of Suppressive Signals within the Drosophila Circadian Neural Circuit Generates Sequential Daily Outputs. *Neuron* **94**, 1173-1189.e4 (2017).

21. F. Port, *et al.*, A large-scale resource for tissue-specific CRISPR mutagenesis in Drosophila. *eLife* **9**, e53865 (2020).
